# Combined Home Exercise Is More Effective Than Range-of-Motion Home Exercise in Patients with Ankylosing Spondylitis: A Randomized Controlled Trial

**DOI:** 10.1155/2014/398190

**Published:** 2014-09-07

**Authors:** Lin-Fen Hsieh, Chih-Cheng Chuang, Ching-Shiang Tseng, James Cheng-Chung Wei, Wei-Chun Hsu, Yi-Jia Lin

**Affiliations:** ^1^Department of Physical Medicine and Rehabilitation, Shin Kong Wu Ho-Su Memorial Hospital, Taipei 11101, Taiwan; ^2^School of Medicine, Fu Jen Catholic University, New Taipei City 24205, Taiwan; ^3^Department of Allergy, Immunology and Rheumatology, Shin Kong Wu Ho-Su Memorial Hospital, Taipei 11101, Taiwan; ^4^Division of Allergy, Immunology and Rheumatology, Chung Shan Medical University Hospital, Taichung City 40201, Taiwan; ^5^Institute of Medicine, Chung Shan Medical University, Taichung City 40201, Taiwan; ^6^Graduate Institute of Integrated Medicine, China Medical University, No. 110, Sec. 1, Jianguo N. Road, South District, Taichung City 40201, Taiwan; ^7^Graduate Institute of Biomedical Engineering, National Taiwan University of Science and Technology, Taipei 10607, Taiwan; ^8^National Defense Medical Center, Taipei 11490, Taiwan; ^9^University of Taipei, Taipei 11153, Taiwan

## Abstract

Home exercise is often recommended for management of patients with ankylosing spondylitis (AS); however, what kind of home exercise is more beneficial for patients with AS has not been determined yet. We aimed to compare the effectiveness of combined home exercise (COMB) and range-of-motion home exercise (ROM) in patients with AS. Nineteen subjects with AS completed either COMB (*n* = 9) or ROM (*n* = 10) program. The COMB program included range-of-motion, strengthening, and aerobic exercise while the ROM program consisted of daily range-of-motion exercise only. After exercise instruction, subjects in each group performed home exercise for 3 months. Assessment included cardiopulmonary exercise test, pulmonary function test, spinal mobility measurement, chest expansion, Bath Ankylosing Spondylitis Functional Index (BASFI), and other functional ability and laboratory tests. After exercise, the COMB group showed significant improvement in peak oxygen uptake (12.3%, *P* = 0.008) and BASFI (*P* = 0.028), and the changed score between pre- and postexercise data was significantly greater in the COMB group regarding peak oxygen uptake and BASFI. Significant improvement in finger-to-floor distance after 3-month exercise was found only in the COMB group (*P* = 0.033). This study demonstrates that a combined home exercise is more effective than range-of-motion home exercise alone in aerobic capacity and functional ability.

## 1. Introduction

Ankylosing spondylitis (AS) is a chronic inflammatory disorder mainly involving the sacroiliac joints and spine, although peripheral joints may also be involved. Inflammation of ligament or tendon insertion at the bone (enthesopathy) is also a characteristic finding. The disease can be accompanied by extraskeletal manifestations, such as acute anterior uveitis, aortic incompetence, cardiac conduction defects, fibrosis of the upper lobes of the lungs, neurological involvement, or renal amyloidosis [1]. In a recent report, patients with AS were at increased risk for cardiac morbidity including coronary artery disease [2]. The prevalence of AS is 0.15% to 0.86% [1].

The main biomechanical problems in patients with AS include limitations in spinal and peripheral joint mobility, restriction of chest expansion [3], reduction of vital capacity [4, 5], and deterioration of aerobic capacity [6]. Carter et al. showed that peak oxygen uptake (${\dot{\text{V}}\text{O}}_{2\;\text{peak}}$) was significantly lower (75% of normal) in patients with AS [6].

The main treatment for AS since the 1960s has been medications and exercise to maintain spinal mobility and function [1]. It has been shown that 2–4 weeks of intense inpatient treatment yields significant improvement of mobility and pain and that the benefit may persist for months or years [7, 8]. Because many patients with AS cannot receive inpatient exercise training, many exercises therapies via outpatient department or even home exercise have been conducted. van Tubergen et al. found that patients with AS receiving spa and weekly group therapy (including physical exercise, sports, and hydrotherapy) for 40 weeks showed improvement in functional ability and quality of life [9]. Ince et al. also reported the benefit of multimodal supervised exercise programs [10]. Helliwell et al. randomized 44 patients with AS to receive (a) intensive inpatient physiotherapy, (b) outpatient hydrotherapy and home exercise, or (c) home exercise alone. Both inpatient and hydrotherapy patients reported more subjective improvement; however, at six months, there were no differences in outcomes between the three groups [8]. In 2000, Uhrin et al. also showed that even unsupervised recreational exercise improves pain and stiffness [11]. ASAS/EULAR also suggested that optimal management requires a combination of nonpharmacological and pharmacological treatments, and home exercise was listed in category IIa in evidence of efficacy [12].

Because most patients with AS in our country are employed, inpatient or regular outpatient exercise program may be not feasible for many patients with AS. For most patients with AS, home exercise is more convenient and more likely to be continued for a long period of time. In addition, for patients with AS, previous studies emphasized range-of-motion exercise, and aerobic exercise was often neglected [13]. Literature review also showed that comparing between different home exercise programs in patients with AS has rarely been reported and, as far as we know, has never been reported in oriental population.

The purpose of this study was to compare the effectiveness of combined home exercise (COMB, including range-of-motion, strengthening, and aerobic exercise) and range-of-motion home exercise (ROM) in Taiwanese patients with AS.

## 2. Methods

### 2.1. Participants

Forty-four adult subjects with AS were recruited from the outpatient clinics of allergy-immunology-rheumatology (AIR) and physical medicine and rehabilitation (PM and R) in a private teaching hospital and an AS care group (a society organized by patients with AS in Taiwan). Inclusion criteria were as follows: (1) fulfilling the 1984 modified New York criteria for AS [14]; (2) age between 20 and 65 years; (3) disease in well-controlled condition; and (4) disease lasting for at least 6 months. Exclusion criteria included (1) presence of serious medical conditions or acute febrile disorders; (2) history of arthroplasties or major operations in the knee or hip joints; and (3) severe arthritis or contracture of knee or hip joints which preclude exercise testing with a bicycle. Use of concomitant medications was allowed, and no instructions were given to subjects to alter their daily activity except regarding the prescribed home exercise program.

Participants who met the inclusion criteria were then scheduled for interviews and testing. Before study enrollment, all participants signed a consent form approved by the hospital ethics committee.

**Figure 1:**
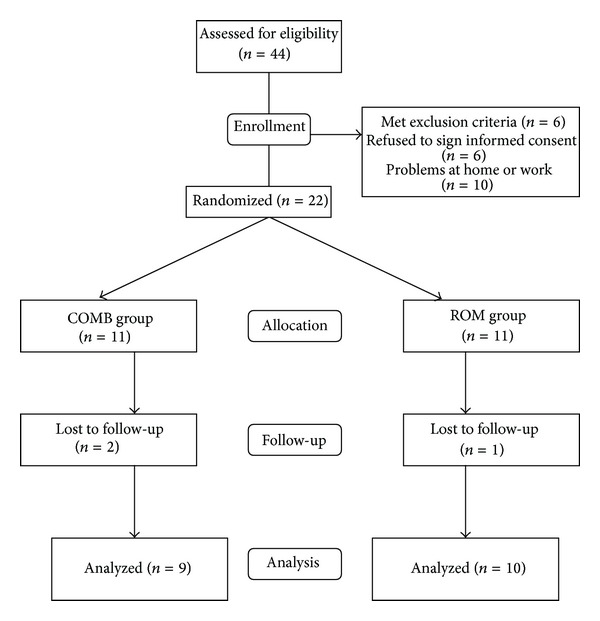
Flowchart for randomization procedure.

Of the 44 subjects with AS screened for the study, 3 had coronary artery disease, 2 had received total hip replacement, 5 refused to sign informed consent, 9 were busy in working or had home problems, and 3 were excluded due to illness or other causes, so that a total of 22 patients were randomized to the 2 home exercise programs. However, 2 in the COMB group and 1 in the ROM group did not complete the study due to personal reasons. Totally, 19 subjects with AS completed the study. Randomization was performed by a computer-generated random-number list. The allocation of the groups was initially concealed in an envelope, which was opened for each consecutive patient to reveal his or her group assignment at the time he or she was recruited into the study. A group of 9 subjects (mean age 36.2 years, standard deviation (SD) 11.7 years) served as the COMB group, and another 10 subjects (mean age 42.1 years, SD 8.8 years) comprised the ROM group (Figure 1).

The demographic data of the study subjects are shown in Table 1. There was no significant statistical difference between the COMB and ROM groups with regard to age, gender, body weight, body height, disease duration, smoking, marriage, exercise habit, and use of medications. None of the subjects participated in regular exercise prior to the study. All subjects were taking nonsteroidal anti-inflammatory drugs (NSAIDs), and most of them were also taking remittive agents. None of the subjects were treated with biologic agents.

### 2.2. Intervention

Subjects in the ROM group received instruction in range-of-motion exercise of the spine and major joints (including the shoulder, elbow, wrist, hip, knee, and ankle) from a senior physical therapist. Chest expansion and breathing exercise were also included. An exercise booklet was also given to each subject. After participants learned how to perform the range-of-motion exercise, they are instructed to conduct exercise at home daily for 3 months. Each range-of-motion exercise was repeated 5 times. Each subject was instructed to perform gentle stretch to tightness at end of the range-of-motion but not to pain.

The COMB group received not only range-of-motion exercise, but also strengthening of the muscles of the major joints (including the cervical spine, thoracolumbar spine, shoulder, elbow, wrist, hip, knee, and ankle) and aerobic exercise (including fast walking, cycling, and swimming as suggested). Each set of strengthening exercises consisted of 10 repetitions, and the intensity was set at 60% to 80% of one repetition maximum [15]. Each subject was asked to perform two sets of strengthening exercises each time, 2 times per week. A rest interval between sets was 2 to 3 minutes. Aerobic exercise program consisted of 5 min stretching of the exercise muscles, 5 min warm-up, 20–30 min aerobic exercise, and 5 min cooling-down. The intensity of aerobic exercise was set between 50% and 80% of ${\dot{\text{V}}\text{O}}_{2\;\text{peak}}\;$ (peak oxygen uptake). Each subject in the COMB group was requested to perform aerobic exercise 3 times per week. The COMB program was also continued for 3 months. Participants in each program were instructed to use daily exercise logs for self-monitoring of the duration, intensity, and frequency of exercise. During the study period, a physical therapist was assigned to monitor the progress of the exercise program by calling each subject every 2 weeks. Compliance with the exercise program was assessed by actual exercise frequency divided by the predicted frequency.

### 2.3. Assessment

Besides background information, spinal mobility (including Schober's test, finger-to-floor distance, occiput-to-wall distance, and range-of-motion of the cervical spine), chest expansion, exercise tolerance test, pulmonary function test, grip strength, Bath Ankylosing Spondylitis Global Score (BAS-G), Bath Ankylosing Spondylitis Functional Index (BASFI), Bath Ankylosing Spondylitis Disease Activity Index (BASDI), erythrocyte sedimentation rate (ESR), C-reactive protein (CRP), and hemoglobin (Hb) were measured at the baseline and immediately after 3-month exercise. Throughout the study, the evaluators did not know the assigned group of each subject. Peak oxygen consumption (${\dot{\text{V}}\text{O}}_{2\;\text{peak}}\;$), finger-to-floor distance, chest expansion, and BASFI were chosen as primary outcome measures according to previous studies [16].

#### 2.3.1. Background Information

Each subject was requested to fill out a self-report data form containing questions about age, gender, body weight, body height, symptom duration, smoking history, marital status, exercise habit, occupational activities, recreational activities, medications, and health history.

#### 2.3.2. Schober's Test

It was an increase in distance between 2 skin marks between the fifth spinal process and 10 cm above from erect standing to maximal forward bending [17].

#### 2.3.3. Finger-to-Floor Distance

It was the shortest distance between fingers and floor on maximal forward flexion of the low back, with knees straight [18].

#### 2.3.4. Occiput-to-Wall Distance

When the patient was standing with buttocks and heels against a wall and trying to touch the wall with the occiput while keeping a horizontal gaze, the distance between the occiput and the wall is measured [18].

#### 2.3.5. Range-of-Motion of the Cervical Spine

Flexion, extension, bilateral rotation, and bilateral side bending of the cervical spine were measured with a special goniometer (CROM) [19].

#### 2.3.6. Chest Expansion

It was measured with a tape at the level of the 4th intercostal space. The difference between maximal inspiration and maximal expiration was calculated [18].

#### 2.3.7. Cardiopulmonary Exercise Test

Exercise tolerance of the subjects was measured by open-circuit spirometry. The test was performed on a bicycle ergometer with the participant in an upright position. It was started with an initial load of 0 watts, with an increment of 10–20 watts/min until exhaustion or appearance of symptoms. BP, ECG, HR, and oxygen saturation were monitored during the test. A physiatrist was present during all testing. Expired gas was analyzed by an automated system instrument (Vmax 29 Cardiopulmonary Exercise Testing Instrument, SensorMedics Corporation, Yorba Linda, California). Variables of exercise tolerance test included HR, BP, oxygen uptake (${\dot{\text{V}}\text{O}}_{2}\;$), metabolic equivalent (MET), work, oxygen pulse, and respiratory exchange ratio (RER) at peak cardiovascular response and at ventilatory threshold (VT) [20, 21].

#### 2.3.8. Pulmonary Function Test

Pulmonary function tests included measurement of the forced vital capacity (FVC), forced expiratory volume in one second (FEV1), FEV1/FVC, peak expiratory flow (PEF), total lung capacity (TLC), residual volume (RV), RV/TLC, and functional residual capacity (FRC).

#### 2.3.9. Grip Strength

It was measured with a hand dynamometer in the dominant hand.

#### 2.3.10. BAS-G

It is a single item question regarding a patient's sense of well-being over the last week and the past six months. The mean of the two scores gives a BAS-G score of 0 (the best) to 10 (the worst) [22].

#### 2.3.11. BASFI

It contains 10 questions assessing activities of daily living and is scored on a 10 cm visual analogue scale (VAS). A final score is obtained by calculating the mean of the 10 items [23].

#### 2.3.12. BASDAI

It consists of 6 questions relating to fatigue, back pain, pain and/or swelling of peripheral joints, localized tenderness, and duration and severity of morning stiffness in the previous week. Each question is answered with a 10 cm VAS and the total score (0 to 10; 0 = the best, 10 = the worst) is calculated according to the instructions [24].

#### 2.3.13. Laboratory Tests

ESR, CRP, and Hb were measured for evaluation of disease activity.

### 2.4. Data Analysis

For demographic data, independent *t*-test or Mann-Whitney *U* test (if distribution was nonnormal) was used for continuous variables, and chi-square test or Fisher's exact test was performed for categorical variables. For between-group comparison, independent-sample *t*-tests were conducted to investigate if there were any differences in the baseline data as well as the changed score between the baseline and the postexercise data between the COMB and the ROM groups. When the assumption of normality or equality of variance was not met, Mann-Whitney *U* test was performed instead. For within-group comparison, we used paired-sample *t*-test or Wilcoxon signed-rank test if the assumption of normality was not met to evaluate whether postexercise data was significantly different from the baseline data in either the COMB or the ROM group. Based on two independent-sample groups (mean differences and their variances) with *α* = 0.05, 2 tails, and sample size of each group being 9 and 10, respectively, powers were calculated. The power was sufficient for occiput-to-wall distance (98%), cervical rotation to the left (97%), Schober's test (92.9%), ${\dot{\text{V}}\text{O}}_{2}\;$% of standard (98%), FEV1/FVC (98%), and BASFI (95%). Statistical analyses were performed using SPSS 15.0 for Windows. A value of *P* < 0.05 was used as an indicator of statistical significance.

## 3. Results

The mean compliance with the exercise program in the COMB group was 48%, while the ROM group had a mean compliance with exercise of 54%. There was no significant statistical difference in compliance between the two groups.

In comparison of spinal range-of-motion and chest expansion, no significant statistical differences between the COMB and ROM groups at baseline were observed except for cervical extension (Table 2), which was more limited in the ROM group (*P* < 0.05). Within-group comparison between baseline and postexercise showed significant improvement in finger-to-floor distance only in the COMB group (*P* = 0.033). However, there was no significant difference between the COMB and ROM groups with regard to changed score between the baseline data and the postexercise data in the spinal range-of-motion and chest expansion.

The cardiopulmonary exercise variables at baseline and after 3-month exercise in both the COMB and the ROM groups are shown in Table 3. There was no significant difference in the baseline data of cardiopulmonary exercise test between the two groups. For within-group comparison of exercise tolerance test variables, significant improvement regarding ${\dot{\text{V}}\text{O}}_{2}\;$, ${\dot{\text{V}}\text{O}}_{2}$ of standard, metabolic equivalent (MET), and HR at peak cardiovascular response and ${\dot{\text{V}}\text{O}}_{2}$, MET, and HR at ventilatory threshold were found in the COMB group; however, significant reduction of ${\dot{\text{V}}\text{O}}_{2}$, ${\dot{\text{V}}\text{O}}_{2}$% of standard, and MET at the peak cardiovascular response and increase of resting HR were found in the ROM group. On comparison of the changed scores between the baseline data and after 3-month exercise data, the COMB group displayed significantly greater improvement in terms of ${\dot{\text{V}}\text{O}}_{2}$, ${\dot{\text{V}}\text{O}}_{2}$ of standard, and MET at peak cardiovascular response and ${\dot{\text{V}}\text{O}}_{2}$, MET at ventilatory threshold.

Table 4 displays comparison of pulmonary function test between the two groups. Either at baseline or after 3-month exercise, no significant difference was observed between the two groups in terms of variables of pulmonary function test. For within-group comparison of the exercise effect, no significant statistical difference was demonstrated in each group except for peak expiratory flow (PEF) in the COMB group. For between-group comparison regarding the changed score between the baseline data and the postexercise data, no significant statistical difference was observed in all of the pulmonary function test data.

On the follow-up of disease activity and functional ability, no significant statistical difference was found between the two groups in ESR, CRP, Hb, grip strength, BAS-G, BASFI, and BASDAI, either at baseline or after 3-month exercise (Table 5). Within-group comparison showed significant improvement (*P* = 0.028) in BASFI after 3-month exercise program (Table 5) only in the COMB group. Between-group comparison also demonstrated significant statistical difference (*P* = 0.041) in changed score of BASFI between the baseline data and postexercise data, in favor of the COMB group.

## 4. Discussion

Our study showed that Taiwanese patients with AS participating in combined home exercise (range-of-motion, strengthening, and aerobic exercise) could improve aerobic capacity as well as BASFI. In this study, the average improvement rate in ${\dot{\text{V}}\text{O}}_{2\;\text{peak}}$ was about 12%. On the contrary, patients with AS in the ROM group had some decrease in ${\dot{\text{V}}\text{O}}_{2\;\text{peak}}$. However, one subject in the ROM group had anemia (Hb = 9.0 gm/dL) for unknown reason, two reduced physical activities due to too much engagement in working, and none of the subjects in the ROM group participated in aerobic or strengthening exercise, which could partly explain the cause of aerobic capacity reduction.

For exercise prescription in patients with AS, previous studies emphasized range-of-motion exercise and posture instructions [13, 25]. The health-related components of physical fitness include aerobic fitness, muscle strength and endurance, flexibility, and body composition. For promoting physical fitness, exercise components usually consisted of aerobic, muscle strengthening, and range-of-motion or stretching exercise [26]. Also, increased cardiovascular morbidity and mortality in patients with AS have been reported [2]. For these reasons, we think that exercise for patients with AS should include aerobic component and muscle strengthening as well as range-of-motion exercise.

Measurement of spinal range-of-motion and chest expansion did not show significant improvement except for finger-to-floor distance in the COMB group (Table 2). Although previous studies demonstrated that participation of patients with AS in 3 to 4 weeks of intensive physiotherapy sessions could help to increase chest expansion, finger-to-floor distance, thoracolumbar rotation, and lateral trunk flexion [16, 27, 28], the studies were done on an inpatient, and usually the number of patients able to attend intensive 3- to 4-week inpatient training program was very limited. Uhrin et al. found that unsupervised recreational exercise with duration more than 200 minutes per week could reduce the severity and pain in patients who had AS for 15 years or less [11]. In Russell's study, a single exercise session induced a small but significant increase in lumbar extension for the vigorous exercise group but no significant change for moderate exercise or nonexercise group [27]. In AS patients with long duration, severe contracture or fusion of spinal and peripheral joints was frequently present, and gentle range-of-motion was not effective in improving the mobility of joints. In our study, both groups of patients with AS had average duration more than 11 years (especially in the ROM group), and fusion or severe contracture in the spine was present in some patients; besides, home exercise may be too gentle to induce change in the range-of-motion of the spine and peripheral joints.

Another cause of no improvement in spinal range-of-motion may be due to low compliance. As has been reported previously, the compliance for home exercise is between 30 and 90%, usually in the lower range [21]. Generally speaking, the compliance with inpatient exercise is highest, followed by supervised outpatient or organized exercise program, and home-based exercise is the lowest. However, in a study by Lim et al., an 8-week home-based exercise program increased joint mobility and functional capacity and decreased pain and depression in patients with AS [29]. In that study, the researchers monitored the patients by telephone every day. Because of lack of manpower, we monitored the patients by phone only once in 2 weeks. If we could have monitored the patients more frequently, the compliance would have been increased, and the effect of exercise might also have been improved. A home-based exercise program is cheaper, time-saving, and more easily accessible to patients. It might still be an effective intervention for patients with AS if the compliance with exercise could be improved.

Our study showed no significant difference between baseline and postexercise data for most of the pulmonary function tests except for PEF in the COMB group (Table 4). No improvement in VC, FVC, and other pulmonary function tests after exercise could partly be reflected by the nonsignificant change in chest expansion (Table 2). Viitanen et al. conducted a 3- to 4-week inpatient training and showed that average increase in VC was 200 mL in men and 270 mL in women [28]. However, at 15-month follow-up after the training, both chest expansion and vital capacity had significantly deteriorated from the baseline [30]. Tomlinson et al. also reported significant improvement in mobility, posture, and lung function from 3-week intensive inpatient physiotherapy [31]. Again, difference in the outcome could be explained by different exercise program (home exercise versus inpatient physiotherapy).

In our study, significant improvement in the functional ability after home exercise was observed only in the COMB group for BASFI (Table 5). Previous study has also shown that BASFI is sensitive to the functional change in patients with AS [22, 29]. van Tubergen et al. also found that combined spa therapy and exercise in addition to medications and physical therapy was associated with significant improvement in BASFI [9]. Another report from Sweeney et al. also demonstrated that a home-based exercise intervention showed a trend for improvement in BASFI [32]. Our study was consistent with those previous reports.

The strengths of this study are as follows. (1) It was prospective, randomized, and blinded to the evaluators. (2) Exercise intervention study in Taiwanese patients with AS has never been reported before. (3) Comparison of different home exercise programs has rarely been reported, even in the Western population. (4) The baseline and postexercise evaluations were very extensive, including aerobic capacity, pulmonary function, range-of-motion of the spine, chest expansion, functional ability, and other disease-related measures. However, our study has some limitations, First, the sample size of 9 or 10 in each group was small, and the statistical power for chest expansion (21%), finger-to-floor distance (40.2%), MET at peak cardiovascular response (12%), and FVC (11.5%) was low. More cases are needed for evaluating change of spinal range- of- motion and pulmonary function test. Secondly, the study did not provide long-term follow-up (e.g., 6 months, 1 year), and thus we did not know whether the exercise effects would be maintained after a long exercise program. Thirdly, we monitored patients once in 2 weeks. A more frequent monitoring by phone or other means might increase the exercise effect. Fourthly, the average duration of disease in patients with AS was more than 11 years, and some patients had severe contracture or fusion of the spinal joints. If we could select patients with shorter duration or with a more flexible spine, the exercise effect might be improved.

## 5. Conclusions

This study demonstrated that a 3-month home-based combined (aerobic, strengthening, and range-of-motion) exercise program significantly improved aerobic capacity and functional ability (BASFI) in patients with AS and was superior to home-based range-of-motion exercise alone. Exercise prescription for patients with AS should include range-of-motion (or stretching), strengthening, and aerobic components and should be started in the early stage of the disease.

## Figures and Tables

**Table 1:** Demographic data of the study subjects.

Characteristic	COMB group	ROM group	*P* value∗
	(*n* = 9)	(*n* = 10)	
Age, years, mean (SD)	36.2 ± 11.7	42.1 ± 8.8	0.219
Gender (M/F)	6/3	7/3	1.000
BW, kg, mean (SD)	64.0 ± 12.0	63.5 ± 9.9	0.935
BH, cm, mean (SD)	164.1 ± 7.8	160.5 ± 8.4	0.461
BMI, kg/m^2^, mean (SD)	23.8 ± 7.8	24.7 ± 3.7	0.462
Disease duration, years, mean (SD)	11.1 ± 6.8	17.3 ± 10.7	0.164
Smoking (yes/no)	1/8	3/7	0.582
Marriage (yes/no)	5/4	5/5	1.00
Regular exercise (yes/no)	0/9	0/10	1.00
Medication			
NSAID (yes/no)	9/0	10/0	1.00
DMARD (yes/no)	6/3	7/3	1.00

COMB: combined home exercise; ROM: range-of-motion home exercise; BW: body weight; BH: body height; BMI: body mass index; NSAID: nonsteroidal anti-inflammatory drug; DMARD: disease-modifying agent. *P values for differences in the baseline data between the COMB and the ROM groups.

**Table 2:** Comparison of chest expansion and spinal range of motion at the baseline and after 3-month exercise between combined home exercise (COMB) group and range-of-motion home exercise (ROM) group.

Measures	COMB group (*n* = 9)			ROM group (*n* = 10)			Both groups
	Baseline mean (SD)	Postexercise mean (SD)	Within-group *P value	Baseline mean (SD)	Postexercise mean (SD)	Within-group ^†^ *P* value	Between-groups ^‡^ *P* value
Schober's test	3.3 ± 2.0	3.8 ± 2.3	0.260	1.4 ± 1.1	2.2 ± 2.0	0.092	0.567
Finger-to-floor distance, cm	19.9 ± 13.8	14.9 ± 12.7	0.033	28.2 ± 12.3	25.4 ± 12.8	1.000	0.141
Chest expansion, cm	2.4 ± 1.6	2.6 ± 0.8	0.553	1.7 ± 2.1	2.5 ± 1.9	0.059	0.391
Occiput-to-wall distance, cm	4.9 ± 5.1	2.7 ± 5.9	0.223	9.7 ± 9.2	9.8 ± 9.4	0.680	0.665
C-ext, degree	41.9 ± 17.7	44.9 ± 23.8	0.596	23.7 ± 17.7∗	28.7 ± 25.9	0.497	0.967
C-flex, degree	37.4 ± 19.3	37.8 ± 18.7	1.000	23.7 ± 15.8	26.4 ± 17.4	0.400	0.615
C-LR, degree	48.1 ± 28.1	53.3 ± 25.0	0.236	32.5 ± 26.4	35.2 ± 21.9	0.482	0.836
C-RR, degree	45.9 ± 26.2	52.6 ± 24.6	0.202	30.0 ± 27.4	34.8 ± 24.9	0.778	0.681
C-LSB, degree	27.1 ± 17.2	31.9 ± 21.5	0.446	18.6 ± 17.9	17.6 ± 17.9	0.610	0.283
C-RSB, degree	33.8 ± 20.9	34.1 ± 22.2	0.779	16.2 ± 17.6	18.6 ± 19.0	0.113	0.362

C-ext: cervical extension; C-flex: cervical flexion; C-LR: left rotation of the cervical spine; C-RR: right rotation of the cervical spine; C-LSB: left side bending of the cervical spine; C-RSB: right side bending of the cervical spine. *P values for differences in the baseline data between the COMB and the ROM groups; ^†^ *P* values for differences in the postexercise data between the COMB and the ROM groups; ^‡^ *P* values for differences in the changed score between the baseline and the postexercise data between the COMB and the ROM groups.

**Table 3:** Comparison of the exercise tolerance variables at the baseline and after 3-month exercise between combined home exercise (COMB) group and range-of-motion home exercise (ROM) group.

Variables	COMB group (*n* = 9)			ROM group (*n* = 10)			Both groups
	Baseline mean (SD)	Postexercise mean (SD)	Within-group *P value	Baseline mean (SD)	Postexercise mean (SD)	Within-group ^†^ *P* value	Between-groups ^‡^ *P* value
Resting state							
HR (beats/min)	77.3 ± 8.7	82.6 ± 11.0	0.173	75.0 ± 7.4	81.3 ± 10.5	0.036	0.870
SBP (mmHg)	120.0 ± 8.7	113.9 ± 6.6	0.155	120.2 ± 13.6	110.6 ± 12.6	0.139	0.595
DBP (mmHg)	73.4 ± 7.4	72.6 ± 4.1	0.905	72.8 ± 7.5	73.1 ± 10.1	0.959	0.775
SpO_2_%	96.1 ± 0.8	96.0 ± 0.9	0.655	95.9 ± 1.3	95.9 ± 1.3	1.000	0.931
Peak response							
$\dot{\text{V}}$O_2_ (mL/kg/min)	20.4 ± 4.2	22.9 ± 4.2	0.008	20.1 ± 5.5	17.9 ± 4.3	0.032	0.001
$\dot{\text{V}}$O_2_% of standard	53.3 ± 8.0	60.1 ± 8.0	0.008	56.2 ± 10.7	50.7 ± 9.1	0.024	0.001
MET	5.8 ± 1.2	6.5 ± 1.2	0.008	5.7 ± 1.6	5.1 ± 1.2	0.032	0.001
Work (W)	124.3 ± 22.2	131.3 ± 31.8	0.138	118.4 ± 34.2	111.4 ± 30.0	0.139	0.055
O_2_ pulse	8.5 ± 2.4	9.0 ± 2.3	0.075	8.3 ± 2.3	7.9 ± 2.2	0.221	0.027
HR (beats/min)	155.1 ± 14.2	162.6 ± 16.4	0.033	152.7 ± 17.2	147.7 ± 21.6	0.474	0.060
SBP (mmHg)	177.0 ± 26.2	178.1 ± 16.7	0.906	182.9 ± 21.7	179.4 ± 20.5	0.221	0.806
DBP (mmHg)	93.9 ± 6.3	97.3 ± 10.1	0.259	95.0 ± 9.6	98.1 ± 12.3	0.507	0.712
RER	1.09 ± 0.9	1.07 ± 0.11	0.261	1.08 ± 0.3	1.06 ± 0.05	0.358	0.967
Ventilatory threshold							
$\dot{\text{V}}$O_2_ (mL/kg/min)	11.0 ± 2.5	12.3 ± 2.2	0.021	11.8 ± 2.8	11.2 ± 3.4	0.415	0.041
MET	3.2 ± 0.7	3.5 ± 0.6	0.021	3.4 ± 0.9	3.2 ± 1.0	0.414	0.049
Work (W)	68.2 ± 24.7	69.7 ± 22.8	0.285	64.4 ± 20.6	60.8 ± 26.0	0.359	0.252
HR (beats/min)	117.8 ± 10.5	123.8 ± 15.0	0.037	119.2 ± 13.6	117.0 ± 18.8	0.541	0.093

HR: heart rate; SBP: systolic blood pressure; DBP: diastolic blood pressure; SpO_2_: peripheral oxygen saturation; $\dot{\text{V}}$O_2_: oxygen uptake; $\dot{\text{V}}$O_2_% of standard: ratio in percentage of oxygen consumption over a standard oxygen uptake; RER: respiratory exchange ratio; MET: metabolic equivalent. *P values for differences in the baseline data between the COMB and the ROM groups; ^†^ *P* values for differences in the postexercise data between the COMB and the ROM groups; ^‡^ *P* values for differences in the changed score between the baseline and the postexercise data between the COMB and the ROM groups.

**Table 4:** Comparison of pulmonary function test at the baseline and after 3-month exercise between combined home exercise (COMB) group and range-of-motion home exercise (ROM) group.

Variables	COMB group (*n* = 9)			ROM group (*n* = 10)			Both groups
	Baseline mean ± SD	Postexercise mean ± SD	Within-group *P value	Baseline mean (SD)	Postexercise mean (SD)	Within-group ^†^ *P* value	Between-groups ^‡^ *P* value
FVC (L)	3.2 ± 0.8	3.1 ± 1.0	0.594	3.3 ± 0.8	3.0 ± 1.0	0.333	0.369
FEV1 (L)	2.9 ± 0.7	2.5 ± 0.8	0.674	2.9 ± 0.7	2.5 ± 0.8	0.766	0.624
FEV1/FVC (%)	89.2 ± 6.2	83.1 ± 7.0	0.811	88.2 ± 6.0	84.7 ± 6.3	0.473	0.364
PEF (L/sec)	7.6 ± 2.3	7.2 ± 2.1	0.021	8.8 ± 1.6	8.0 ± 2.4	0.074	0.514
VC (L)	3.2 ± 0.8	3.1 ± 1.0	0.515	3.3 ± 0.9	3.1 ± 1.0	0.610	0.327
TLC (L)	5.0 ± 1.0	4.9 ± 0.9	0.767	5.1 ± 1.0	5.0 ± 1.0	0.878	0.744
RV (L)	1.7 ± 0.6	1.8 ± 0.4	0.859	1.7 ± 0.6	1.9 ± 0.5	0.506	0.653
RV/TLC (%)	35.0 ± 10.3	38.1 ± 10.8	0.812	34.4 ± 10.4	39.2 ± 10.2	0.540	0.486
FRC (L)	3.2 ± 0.7	3.1 ± 0.8	0.678	3.1 ± 0.7	3.2 ± 0.7	0.683	0.568

FVC: forced vital capacity; FEV1: forced expiratory volume at 1 second; PEF: peak expiratory flow; VC: vital capacity; TLC: total lung capacity; RV: residual volume; FRC: functional residual capacity. *P values for differences in the baseline data between the COMB and the ROM groups; ^†^ *P* values for differences in the postexercise data between the COMB and the ROM groups; ^‡^ *P* values for differences in the changed score between the baseline and the postexercise data between the COMB and the ROM groups.

**Table 5:** Comparison of grip strength, functional ability, and disease activity variables at the baseline and after 3-month exercise between combined home exercise (COMB) group and range-of-motion home exercise (ROM) group.

Variables	COMB group (*n* = 9)			ROM group (*n* = 10)			Both groups
	Baseline mean (SD)	Postexercise mean (SD)	Within-group *P value	Baseline mean (SD)	Postexercise mean (SD)	Within-group ^†^ *P* value	Between-groups ^‡^ *P* value
Grip strength (kg)	28.6 ± 11.0	30.5 ± 12.0	0.109	29.5 ± 10.7	31.1 ± 9.2	0.262	0.682
BAS-G (0–10)	5.6 ± 2.7	3.6 ± 2.0	0.085	5.0 ± 2.8	4.1 ± 3.5	0.285	0.567
BASFI (0–10)	3.7 ± 3.3	1.9 ± 2.3	0.028	3.5 ± 2.9	3.5 ± 3.1	0.859	0.041
BASDI (0–10)	4.2 ± 1.9	3.7 ± 1.8	0.441	4.5 ± 2.1	4.5 ± 3.0	0.953	0.414
ESR (mm/h)	36.8 ± 28.6	24.8 ± 12.0	0.343	24.7 ± 23.1	25.0 ± 28.3	0.673	0.743
CRP (mg/dL)	1.27 ± 1.10	0.79 ± 0.56	0.260	1.07 ± 1.24	0.9 ± 0.99	0.345	1.000
Hb (gm/dL)	14.1 ± 1.1	14.1 ± 0.9	0.812	14.2 ± 1.8	14.1 ± 2.3	0.683	0.653

BAS-G: Bath Ankylosing Spondylitis Global Score; BASFI: Bath Ankylosing Spondylitis Functional Index; BASDI: Bath Ankylosing Spondylitis Disease Activity; ESR: erythrocyte sedimentation rate; CRP: C-reactive protein; Hb: hemoglobin. *P values for differences in the baseline data between the COMB and the ROM groups; ^†^ *P* values for differences in the postexercise data between the COMB and the ROM groups; ^‡^ *P* values for differences in the changed score between the baseline and the postexercise data between the COMB and the ROM groups.
